# Editorial: Evaluation of fitness in stroke survivors

**DOI:** 10.3389/fstro.2025.1682445

**Published:** 2025-08-29

**Authors:** Felipe Cunha, Arthur de Sá Ferreira, Adrian Wayne Midgley

**Affiliations:** ^1^Laboratory of Physical Activity and Health Promotion, Institute of Physical Education and Sports, Rio de Janeiro State University, Rio de Janeiro, Brazil; ^2^Laboratory of Computational Simulation and Modeling in Rehabilitation, Postgraduate Program in Rehabilitation Sciences, Centro Universitário Augusto Motta, Rio de Janeiro, Brazil; ^3^Department of Sport & Physical Activity, Edge Hill University, Ormskirk, United Kingdom

**Keywords:** stroke, recovery, rehabilitation, cardiorespiratory fitness, cardiopulmonary exercise test, balance, body composition, muscular fitness

Stroke remains a leading cause of long-term disability worldwide, often resulting in impairments in cardiorespiratory and neuromuscular function (Tsao et al., 2023). These limitations contribute to decreased physical activity, increased fatigue, and heightened sedentary behavior, jeopardizing functional independence and increasing the risk of recurrence. This Research Topic showcase recent advances in evaluating key domains of physical fitness in stroke survivors, including cardiorespiratory fitness, muscular strength, and endurance, neuromotor control, fatigue tolerance, and body composition.

Cardiorespiratory fitness assessment remains a cornerstone of post-stroke evaluation. The cardiopulmonary exercise test (CPET) is recognized as the gold standard for determining maximal oxygen uptake (VO_2max_), however, its validation, safety, and feasibility in stroke populations remain underexplored. Qu et al. addressed this gap by examining the decline in cardiorespiratory fitness post-stroke using resting-state functional magnetic resonance imaging, opening new perspectives for combining physiological and neuroimaging data in this population.

Accurate assessment of neuromuscular function and physical performance is equally critical. Pu et al. developed a nomogram to predict sarcopenia risk in stroke patients, incorporating anthropometric and biochemical markers, while Zhong et al. validated a Chinese version of the performance-oriented mobility assessment, ensuring reliability for use in chronic stroke survivors. Bi et al. further linked serum albumin levels to severe impairment in activities of daily living (ADLs), reinforcing the role of nutritional and metabolic markers in functional prognosis.

The interplay between physical health, psychological status, and functional independence also emerged as a key theme. Dan et al. demonstrated how depression mediates the link between stroke and fracture risk, highlighting the need for integrative assessments that include emotional and cognitive domains. Similarly, Lin and Liu proposed a predictive model for ADL dysfunction, offering clinicians a tool to anticipate limitations early in the recovery process.

Contributions addressed innovative assessment and rehabilitation strategies. Bian et al. performed a network meta-analysis comparing different physical stimulation therapies, offering evidence to guide upper limb motor rehabilitation strategies. Dai et al. explored the concept of exercise preference in stroke survivors, emphasizing the value of patient-centered approaches when designing fitness evaluations and rehabilitation plans.

Lastly, Yin et al. analyzed thrombectomy timing by stroke subtype, and Chunjuan et al. applied machine learning clustering to inflammatory profiles, both enhancing our understanding of physiological factors influencing recovery potential.

These studies represent a multidisciplinary effort to improve the precision, relevance, and personalization of fitness assessment in stroke rehabilitation. Continued research must ensure that tools are accessible, scalable, and responsive to the specific needs of stroke survivors across the recovery continuum. We hope this Research Topic inspires further innovation and collaboration in optimizing fitness assessment and rehabilitation strategies in stroke care.
